# Oral lesions as the presenting manifestation of syphilis: A case series of an alarming trend in Kuwait

**DOI:** 10.1016/j.idcr.2025.e02338

**Published:** 2025-08-05

**Authors:** Anwar A. AlMuzaini, Dalal H. AlOmar, Jassem M. Bastaki

**Affiliations:** a Al-Adan Dental Specialty Center, Ministry of Health, Kuwait; b Jaber Al-Ahmad Dental Specialty Center, Ministry of Health, Kuwait; c Al-Sabah Hospital, Ministry of Health, Kuwait

**Keywords:** Syphilis, Kuwait, Oral, STD

## Abstract

Syphilis is a sexually transmitted bacterial infection caused by the spirochete *Treponema pallidum*. The global disease burden has recently been affected by a rise in the incidence and prevalence of syphilis across different regions. The latest epidemiological data available on the incidence of syphilis in Kuwait dates back to the 20th century, with no additional available data published since. While the signs and symptoms of syphilis may vary depending on the stage of infection, the oral cavity is typically affected at the primary and/or secondary stages. The diagnosis of syphilis can be difficult without suspicion, as it is known to mimic other diseases. In this case series, we present cases where oral lesions were the first, and in the majority of cases, were the only presentation of secondary syphilis. Most patients were initially misdiagnosed, resulting in a delay in their management. Healthcare providers need to be aware of the various oral manifestations of syphilis, as it can be the first and only presenting sign of the disease. Identifying oral lesions is key in diagnosing syphilis and preventing further transmission and complications of the disease. Diagnosis is made through serologic testing or direct detection of *Treponema pallidum* in tissues. The stage at diagnosis, as well as neurologic involvement, determines the treatment.

## Introduction

Syphilis is caused by the bacterial pathogen *Treponema pallidum* primarily through sexual or vertical transmission [1]. Syphilis was first recognized at the end of the 15th century [2], [3]. The incidence decreased with the development of penicillin; however, there has been a recent increase in cases globally [4], [5]. According to the World Health Organization (WHO), approximately 8 million adults between the ages of 15 and 49 were infected with syphilis in 2022 [6]. Except for a few studies that primarily discuss extra-oral manifestations, there is no recent epidemiological data available on syphilis in Kuwait [7], [8], [9]. This multicenter study reports a case series of syphilis in Kuwait with oral lesions as the presenting sign of the disease. The aim is to highlight the importance of healthcare professionals in appropriately identifying and diagnosing these lesions.

## Case-series

**Fig. 1:**
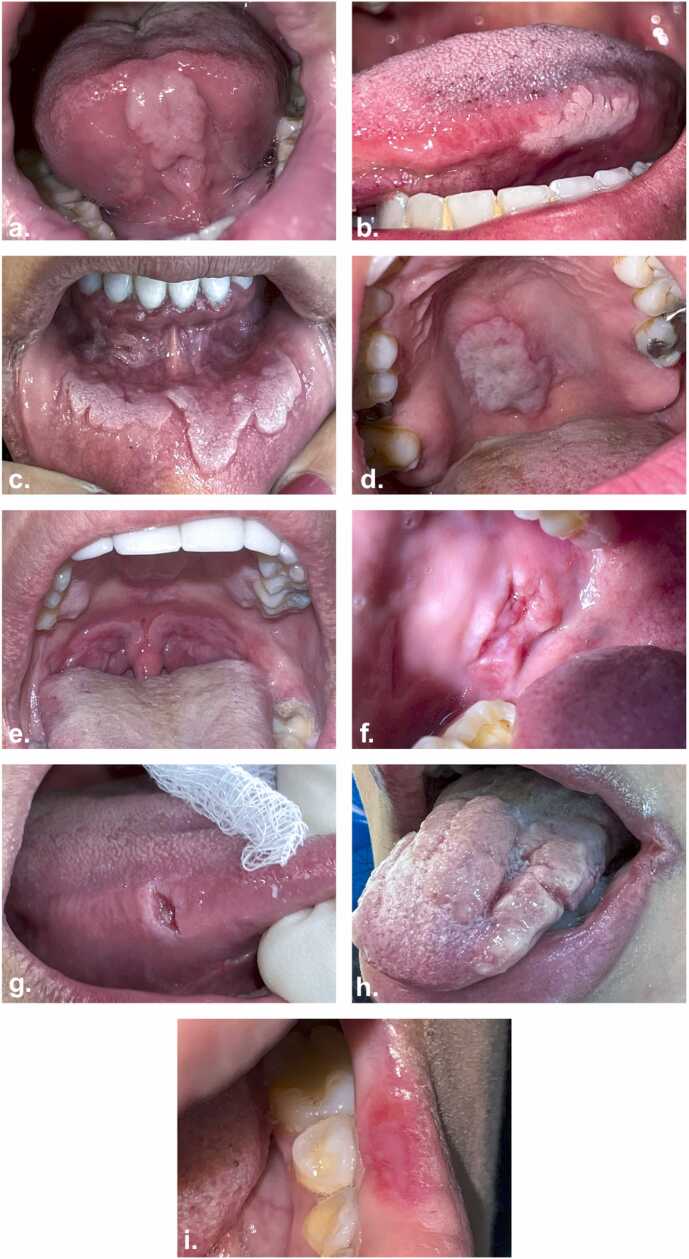
Various oral manifestations of secondary syphilis. Mucous patches (a–e), ulcerative (f,g), papular (h), and macular (i).

Over a three-year period, from 2021 to 2024, 32 patients with various oral complaints were referred to oral medicine clinics at five different specialized dental centers, covering all governorates of Kuwait, within Kuwait’s Ministry of Health. Patients ranged in age from 14 to 48 years, with 20 males and 12 females (Table 1). The main route of infection was heterosexual transmission. Patients complained of generalized oral cavity and oropharyngeal lesions, some of which were painful. Dentists and otolaryngologists initially evaluated patients with various unsuccessful attempts to treat the lesions. Patients were then referred to oral medicine specialists. Clinically, patients showed diverse oral mucosal changes with three distinctive features of secondary syphilis: mucous patches, ulcerations, and maculopapular lesions. All patients presented with multiple, slightly raised, white mucous patches of approximately 1–2 cm in size on various sites of the oral cavity, including upper and/or lower labial mucosa, lip commissures, buccal mucosa, tongue, and hard palate (Fig. 1a–d). Among those, 12 presented with bilateral round patches that were approximating, flat to slightly raised, red and/or white on the posterior wall of the oropharynx (Fig. 1e). Only one patient presented with ulceration of the right lateral tongue and buccal mucosa (Fig. 1f–g). Papular lesions were noted on the posterior two-thirds of the dorsal and lateral surface of the tongue in 3 cases (Fig. 1h). A macular lesion was noted on the lower labial mucosa in one case (Fig. 1i). Except for two patients who presented with systemic findings, a single 14-year old female and a married 38-year old female, oral lesions were the only clinical manifestation of the disease. Systemic findings included malaise, anorexia, weight loss, and severe hair loss. There were no signs or symptoms of congenital, primary, or tertiary syphilis.

**Table 1:** Demographic data of patients.

	Number of patients(N = 32)
**Sex**	
Male	20
Female	12
**Age**	
10–19	2
20–29	13
30–39	11
40–49	6
**Marital Status**	
Single	16
Married	15
Divorced	1

Due to its mimicking nature and various manifestations, clinicians performed oral biopsies on the first twenty-one patients who presented with the lesions to arrive at the correct diagnosis. All specimens exhibited a general morphological manifestation, with confounders, adding to the complexity of this notorious mimicker. Generally, there was microscopic evidence of chronic submucosal inflammation coupled with superficial mucosal acute neutrophilic infiltrate. This peculiar pattern varied in the degree of manifestation across all cases and between the different biopsies from the same patient. With clinical and/or histologic suspicion of syphilis, treponema immunohistochemical (IHC) testing was performed immediately, supporting the diagnosis in all biopsied cases at the histopathologic level. All 32 patients were screened with Rapid Plasma Reagin (RPR) and Venereal Disease Research Laboratory (VDRL) tests. Treponema pallidum hemagglutination assay (TPHA) serological testing then confirmed the diagnosis of syphilis in all patients. All cases were reported to the nearest public health office. Patients were screened for other sexually transmitted diseases (STD), including human immunodeficiency virus (HIV), gonorrhea, chlamydia, hepatitis B and C, instructed on STD prevention strategies, and received treatment at Kuwait Infectious Disease Hospital. Treatment consisted of intramuscular injections of Penicillin G Benzathine once a week for 3 weeks or alternatively doxycycline 100 mg twice daily for 28 days, resulting in the resolution of all oral lesions.

## Discussion

*Treponema pallidum* causes syphilis as a result of direct skin or mucosal inoculation. Dissemination occurs through the bloodstream and lymphatics following host cell attachment. There is a 9–90-day incubation period after which clinical signs of syphilis are apparent [10]. The disease progresses through four stages, each characterized by unique symptoms and risks if left untreated. Primary syphilis is characterized by regional lymphadenopathy and a painless, ulcerated, and indurated chancre at the site of contact with an infected lesion [11]. Typically, the genital areas are affected, though other sites, including the oral cavity, may be involved. Secondary syphilis develops a few months after and is characterized by a nonpruritic rash, mucosal lesions, fever, and lymphadenopathy, among others [4]. Although anti-treponemal antibodies are formed, spirochetes’ presence is highest and continues to spread during secondary syphilis [10]. If untreated, syphilis will enter a latent stage where the patient is asymptomatic [1]. Tertiary syphilis occurs within 2–50 years of infection with untreated syphilis and presents with various multisystem involvement, including neurologic, cardiovascular, otic, and ocular. Neurosyphilis, however, may occur at any stage [4].

A notable change in the trend of global syphilis incidence has been observed, as recent data suggest an unprecedented rise with an increase in the adult and congenital syphilis rate per 100,000 live births from 2016 to 2022 [6]. There is, however, a paucity of epidemiological studies assessing syphilis in Kuwait. Al-Fouzan et al. (2004) reported an increase in the incidence of syphilis in Kuwait, from 2.3/100,000 in 1992–7.0/100,000 in 1995, with a decline thereafter [12]. A 2007 review of the clinical patterns of STDs in one region in Kuwait, Farwaniya, did not identify any cases [9]. A recent systematic review and meta-analysis assessed the prevalence of syphilis in the Middle East and North Africa region, including Kuwait. Kuwait, however, was classified into the Gulf region alongside five other countries, and the data presented were the pooled prevalence rates of all countries included [13]. To the best of our knowledge, there have been no additional epidemiological studies assessing syphilis in Kuwait.

Oral manifestations of secondary syphilis vary considerably and can be the only clinical presentation of the disease, as seen in most cases discussed in the present case series [14], [15]. The differential diagnosis based on the clinical presentation includes traumatic ulcers, angular cheilitis, aphthous ulcers, leukoplakia, contact mucositis, drug eruption, pharyngitis, and neoplasia. A thorough medical examination, including medical history, review of systems, and physical examination, followed by serological testing, is essential to diagnose syphilis in suspected individuals. Given the societal norms and stigma associated with STDs, most patients denied sexual contact with multiple partners or extramarital relations.

Histologically, the chronic inflammatory cell infiltrate is usually composed predominantly of plasma cells, though it can have varying ratios of plasma cells and lymphocytes. The acutely inflamed mucosa may exhibit hyperplasia with or without atypia, which can be prominent at times, raising concerns about dysplasia. In the event of clinical suspicion with non-specific histological results, serological testing becomes essential.

According to the most recent recommendations for syphilis testing by the Centers for Disease Control and Prevention (CDC), non-treponemal tests such as RPR and VDRL remain helpful screening tools for diagnosing syphilis. Still, reactive quantitative titers must be confirmed by treponemal serological testing. Results are variable and depend on the stage at the time of testing, bacterial load, and prior infection with syphilis, whether treated or untreated. Studies do show high sensitivity, approaching 100 %, of non-treponemal and treponemal tests for diagnosing secondary syphilis [16].

## Conclusion

Oral lesions may be the presenting sign of syphilis, which can present a diagnostic challenge to clinicians as it is thought to be a great mimic. Dentists and other healthcare providers need to be familiar with the different oral clinical presentations of syphilis. If misdiagnosed or left untreated, the consequences can pose a risk to the patient and a public health concern.

## CRediT authorship contribution statement

**Anwar A. AlMuzaini:** Writing – review & editing, Writing – original draft, Data curation, Conceptualization. **Dalal H. AlOmar:** Writing – review & editing, Writing – original draft, Data curation, Conceptualization. **Jassem M. Bastaki:** Writing – original draft, Data curation, Conceptualization.

## Consent

An official informed consent form, as approved by Kuwait’s Ministry of Health, was used to obtain written consent from patients for clinical photography for publication purposes, with identifying information removed to ensure confidentiality and anonymity.

## Ethical approval statement

Written informed consent was obtained from the patient for publication of this case report and accompanying images. A copy of the written consent is available for review by the Editor-in-Chief of this journal on request

## Funding source

None.

## Declaration of Competing Interest

The authors declare that they have no known competing financial interests or personal relationships that could have appeared to influence the work reported in this paper.
